# The National Longitudinal Study of Young Life Scientists: Career differentiation among a diverse group of biomedical PhD students

**DOI:** 10.1371/journal.pone.0234259

**Published:** 2020-06-09

**Authors:** Christine V. Wood, Remi F. Jones, Robin G. Remich, Anne E. Caliendo, Nicole C. Langford, Jill L. Keller, Patricia B. Campbell, Richard McGee

**Affiliations:** 1 Department of Medical Social Sciences, Northwestern University Feinberg School of Medicine, Chicago, IL, United States of America; 2 Scientific Careers Research and Development Group, Northwestern University Feinberg School of Medicine, Chicago, IL, United States of America; 3 University of Arizona College of Medicine (retired), Tucson, AZ, United States of America; 4 Campbell-Kibler Associates, Groton, MA, United States of America; 5 Faculty Affairs, Northwestern University Feinberg School of Medicine, Chicago, IL, United States of America; Charles P. Darby Children's Research Institute, 173 Ashley Avenue, Charleston, SC 29425, USA, UNITED STATES

## Abstract

Young biomedical PhD scientists are needed in a wide variety of careers. Many recent efforts have been focused on revising training approaches to help them choose and prepare for different careers. However, very little is known about how biomedical PhD students decide on and “differentiate” into careers, which limits the development of new training models. This knowledge gap also severely limits efforts to increase the representation of women and some racial/ethnic groups in academic research careers. Previous studies have used cross-sectional surveys of career interests and ratings, and have not been designed to identify career intentions. They also are limited by single-time data and response bias, having typically asked participants to recount decisions made years in the past. This report draws on annual, in-depth interviews with 147 biomedical PhD students from the start of the PhD to graduation. Qualitative content analysis methods were used to fully understand scientific development and career intentions over time. Longitudinal analysis reveals a striking level of fluidity and complexity in career intentions over time. Contrary to previous studies and the dominant narrative, data do not show generalized shifts away from academic careers. In addition to those who are consistent in this intention from the start, nearly as many students shift toward research academic careers as away from them, and only modest differences exist by gender and race/ethnicity. Thus, the dominant narrative misses the high fraction of individuals who acquire or sustain their intention to purse an academic research career during training. Efforts to increase diversity in academia must capitalize on and support those who are still considering and evolve toward an academic career. Efforts to revise research training should incorporate knowledge of the tremendous fluidity in when and how career differentiation occurs.

## Introduction

For more than 30 years, the biomedical research and training communities have faced two major concerns: 1) how to prepare PhD students and postdocs for changing and expanding career roles; and 2) the low levels of gender and racial/ethnic diversity across the workforce, but particularly in academic careers. These two challenges are in many ways distinct, and much time and effort has been devoted to addressing them separately. However, solutions to both have been hampered by a knowledge gap—the absence of in-depth, prospective data on the processes of how biomedical trainees “differentiate” into their chosen careers. This gap is problematic because the U.S. (as well as countries across Europe and Asia) commits a great deal of financial and human resources to train new PhD scientists as science careers evolve and to increase the number of women and historically underrepresented individuals (UR) in academic careers.

In the 1990s, a vigorous national debate emerged on whether the U.S. was producing too many biomedical PhDs as the number of faculty positions was declining relative to the number of new PhD scientists [1]. However, data revealed that the unemployment rate for biomedical PhDs was low, not exceeding 2.4% between 2001 and 2010 [2]. Research also showed that more biomedical PhDs were choosing careers outside of academia [3]. National-level studies and reports have led to proposed changes in PhD training designs to prepare graduates for a variety of careers [4–6], and universities have dedicated resources to career development for multiple roles in science [7]. This “branching” career model has been largely accepted within the biomedical training community [8]. Unfortunately, the evolution of the biomedical careers paradigm has occurred with very limited research on how trainees actually make career decisions and “differentiate” into their intended careers during training. Precise and real-time knowledge of these decisions is necessary to understand how and when to offer guidance for career development and selection.

With respect to the challenge of gender and racial/ethnic diversity, some improvements have been made at the undergraduate and PhD training levels. For example, women comprise more than half of biomedical doctoral degree recipients, but hold less than 40% of full-time faculty positions in biomedicine with very minimal expansion into leadership roles [9]. Even more glaring is the underrepresentation of Black/African-American, Hispanic/Latino, Pacific Islander, and Native American scientists (UR groups) among faculty. In 2015, UR students made up about 8% of doctoral recipients in the biomedical sciences [9], yet their representation among academic faculty lags far behind this figure. A 2014 report revealed that UR scientists held only 2% of tenure-track positions in medical school basic science departments [10], and a 2017 study found that in biology departments across 40 prominent, public research universities in the U.S., Black/African-American scientists held only 0.7% of tenure-track positions [11]. Without detailed knowledge of career decision-making processes, it is impossible to identify why initiatives to diversify faculty have failed and what changes are needed for future efforts.

Studies have provided some insights into the problem of underrepresentation and career access on a broad scale, though previous research is limited in scope. The most widely-cited studies document a trend of declining interest in academic careers which occurs early in PhD training and is most severe for women and UR students [8, 10]. These studies are based on single-time surveys. While illustrative of general tends, these results are limited in their ability to address long-term outcomes among individual students. Additionally, while single-time surveys are valuable for offering snapshots into respondents’ career thinking, they rely on participants’ ability to recount past events, posing major problems of response or information bias. Previous surveys have also relied on vague metrics to measure trainees’ career plans. Metrics such as “interest” in careers [12], “desirability” of careers [13], and “attractiveness” of careers [14] have helped illustrate general trends of career interest and desirability, but they do not reveal the complexity of factors involved in career intention and differentiation. By drawing on longitudinal research with a large and diverse group of biomedical PhD students, this paper begins to fill these gaps in knowledge necessary to address career development needs and faculty diversity within biomedical training and career choice.

This report is based on annual, in-depth interviews with 147 biomedical PhD students from the start of the PhD through graduation. Such an endeavor requires an ambitious and rarely used methodology: large-scale, qualitative longitudinal research following scientists over time. This first report of its kind reveals new insights in the patterns of stability and change in career intentions. The results lay the foundation for deep understanding of factors and mechanisms that lead students to shift toward and away from (i.e., “differentiate”) into careers, including the unexpected observation that almost as many students shift ***toward*** academic careers as ***away*** from them.

## Materials and methods

The National Longitudinal Study of Young Life Scientists (NLSYLS) was initiated in 2008 to better understand the career decision-making processes of a diverse group of aspiring biomedical scientists using annual interviews. Its inception, recruitment of students, interview protocols, and other aspects have been described in detail [15–18]. Between 2008 and 2012, students were recruited in multiple waves from colleges and universities throughout the U.S., including Puerto Rico. Students entered the study either a year or two prior to graduate school (from among those considering PhD training) or at the start of a biomedical PhD program. The 1st and 2nd interviews were conducted in person at campuses. Subsequent ongoing yearly interviews were conducted by telephone. Interviews lasted ~1 hour on average and were recorded, transcribed, and checked for accuracy.

Transcripts were coded thematically using NVivo software. Initial codes were broad and included a range of information from personal histories, family, and academic experiences prior to graduate school; a comprehensive picture of experiences during graduate school; and an annual query into how they envision their professional and personal future. Data for this report are drawn specifically from initial responses to the following questions, along with interviewers’ probing for clarification and expansion:

- What are your plans for immediately after the PhD? Are you considering a post doc or something else? What is influencing your planning?
- Where do you see yourself in 10–15 years?

This study was reviewed and approved by the Northwestern University Institutional Review Board (STU00017678). All participants in the study provided informed consent prior to their first interview.

The research team developed a rubric with specific career categories. Three team members used the rubric with coding reports of career interests, goals, and knowledge to assign career intentions at each interview. To be designated a top career intention, an individual had to have made a clear statement that it was their primary intention and the path they would pursue if they were to begin a career at that time. If an individual was clearly weighing more than one career possibility, they were assigned a mixed career intention. For a career to be designated as ruled out, the student would have stated a clear lack of intent. At least two members of the research team reviewed data from each transcript to identify and concur on assigned career intentions. The top career choice for each participant at each interview time was used to create a visual display of career trajectories over time. This process allowed us to identify a “point of differentiation” for each student, the point at which the career decision did not waver.

## Results

The sample of 147 described in this paper consists of 93 women, 25 of whom are classified as UR (27% of all women or 17% of the sample). It consists of 54 men, 11 of whom are classified as UR (20% of all men or 7% of the sample). These demographics are also described in Table 1.

**Table 1:** Career intentions at graduation.

	Academic Research			Academic Teaching		Industry Research		Other Research		Non-Research Science		Non-Differentiated	
**By Demographics**													
	***Totals*** *(n = 147)*	*39*	*(27%)*	*17*	*(12%)*	*22*	*(15%)*	*9*	*(6%)*	*25*	*(17%)*	*35*	*(23%)*
Underrepresented (UR)	Women (n = 25)	5	(20%)	2	(8%)	0	(0%)	3	(12%)	6	(24%)	9	(35%)
	Men (n = 11)	3	(27%)	2	(18%)	3	(27%)	0	(0%)	1	(9%)	2	(18%)
Well-represented (WR)	Women (n = 68)	18	(26%)	7	(10%)	13	(19%)	5	(7%)	12	(18%)	13	(19%)
	Men (n = 43)	13	(30%)	6	(14%)	6	(14%)	1	(2%)	6	(13%)	11	(26%)
**By Career Intention Over Time**													
***Totals***		*39*		*17*		*22*		*9*		*25*		-	
Unchanging		11	(28%)	8	(47%)	2	(9%)	1	(11%)	0	(0%)	-	
Fluctuating		9	(23%)	3	(18%)	10	(45%)	1	(11%)	0	(0%)	-	
Increasing		19	(49%)	6	(35%)	10	(45%)	7	(78%)	25	(100%)	-	

This table displays: (1) the breakdown of study participants into their primary career intentions at graduation; and (2) patterns of change and stability for intended careers over time. The top half displays career intentions at graduation by gender and UR/WR status. The percentages in the very top row are based on the entire sample, while each descending row gives percentages based on gender and WR/UR status. Using Pearson’s chi-squared tests, there are no significant (p < .05) differences between demographic groups for each primary career intention. The bottom half displays patterns of career intentions over time for each career type. Unchanging means no change in primary career intention from the beginning of the PhD until graduation; fluctuating means the same primary career intention at the start and end of the PhD with vacillation during the PhD; and increasing means a shift towards a primary career intention during the PhD, with limited or no intention indicated at the start. Note, two students who left science after graduation are not included in this table.

Results are displayed and analyzed based on career intentions at each interview during the PhD through graduation. Career categories included academic research (PI), academic teaching (predominantly or completely teaching), industry research, other research (e.g., government research or staff scientist), and non-research science (e.g., science policy or regulatory work). At the time of graduation, only two participants indicated they would leave science entirely, and 35 were undecided on their top choice. Students with more than one top choice are labeled as “non-differentiated” and represented in the figures among those with no top choice.

### Career intention trajectories over time in PhD training

**Fig 1:**
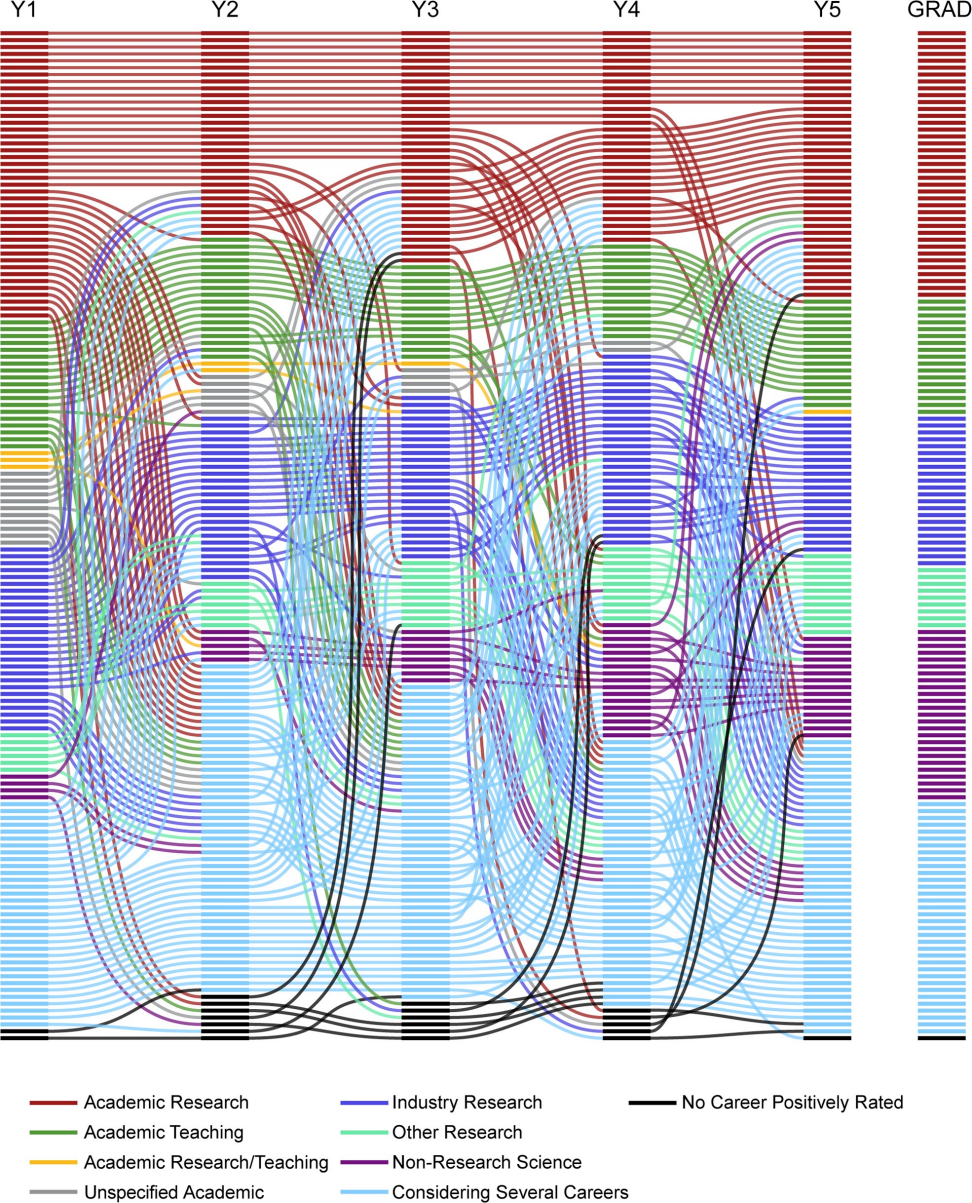
Change of career intention from start to finish of the PhD. This alluvial chart tracks the primary career intentions of 147 participants from the first year of a PhD program through graduation. Each column represents a time point separated by a one-year interval, with “Y1” representing the start of the first year of a PhD program and “Grad” reflecting the year of graduation. Each horizontal line represents an individual, with each color corresponding to a career type. Following a line left to right reveals the trajectory of an individual’s primary career intentions over time. The color of the connecting line between years matches the career intentions of the previous year, allowing for one to observe change even while examining a single column. Since students spent different amounts of time in their respective PhD programs, the Grad year cannot be connected to the rest of the timeline while maintaining the year-by-year quality of the columns. The start of the fifth year of the PhD was the first year in which anyone in the study graduated and is the last time point reflected before the Grad year. Those in “Considering Several Careers” did not indicate a single primary career, instead focusing on two or more possibilities, while those with no primary career intentions are represented with “No Career Positively Rated.”

The alluvial graphic (Fig 1) reveals the complexity and fluidity of career decision-making among students during their PhD training. To be placed in any career category, an individual would have expressed a primary intention towards that career in their annual interview. Changes in primary career intention across years were very common (2.9±1.8 SD changes over the course of PhD training, *n* = 147). The longitudinal data reveal shifts toward and away from all career options, as well as a subset of individuals who did not waver in their intentions from the beginning of the PhD to graduation. The alluvial figure accounts for students whose career choices were “mixed,” meaning they clearly indicated two or more careers as top choices. A very small number of students at each time point indicated no clear career intention, and these cases are reflected in the graphic.

### Career intentions at graduation and stability by career type

Among students who had differentiated by graduation, academic research was the most popular career choice, followed by non-research science and industry research. While previous studies report an overall decline in interest in academic research careers during graduate school, the current data portray a more complex story. In this sample, the number of students intending to pursue academic research careers at the beginning of the PhD (*n* = 42) and at graduation (*n* = 39) were very similar, but the individuals populating this category changed substantially. This observation suggests that students move toward and away from academic careers during the PhD with similar frequencies.

The patterns of movement towards and away from academic research are complex. Table 1 shows that of the 39 individuals intending to pursue academic research at graduation, only 11 were consistent in their intentions from the start of the PhD through graduation. For those with an academic research career intention at graduation, a substantial number of students shifted towards that intention during the PhD. Others fluctuated by starting with an academic research intention, wavering during the PhD, and settling on it at graduation. Nearly half (*n* = 19) of the 39 had shifted towards academic research, despite indicating little or no intention at the beginning. These 19 academic “increasers” were mostly women from various racial/ethnic backgrounds.

While small cell sizes prevented statistical testing, two trends emerged from the data. The first trend was that UR women appeared to be more likely than any other group to shift towards academic research, even though they were least likely overall to select into this career. This finding is especially compelling given the existing data showing decline in interest in academic careers among women and UR students. While a full presentation of interview data is beyond the scope of this paper, case data do help reveal why some participants shifted towards academic research at a late stage in training.

Among those women shifting towards academic research was Leticia (pseudonym), who self-identified as a Black woman. She began her graduate training intending to pursue a career in industry. Though drawn to research, Leticia expressed skepticism of academia in her first interview, stating, “It seems like people in academia are too stressed. So, that’s not my ideal lifestyle…if I wanted a family, then academic wouldn’t be for me.” She echoed these concerns during her PhD, repeatedly mentioning the challenges of work and family as deterrents from academia. By graduation, however, an academic research career had become her first choice. She gave several reasons for her late-stage shift, among them a postdoctoral fellowship in a promising field and the chance to work in the same place as her husband. She explained her reasoning in a series of statements.

“[I like] the control [of academic research]. The control over what I’m doing, control about the environment, the atmosphere, where the research is going.”

“I think [my postdoc field] will be very fundable. I think it is a very hot area.”

“[Universities] like to match couples. They like to see couples well-matched, and the fact that [my husband] is interested in research and I am a researcher helps.”

Like Leticia, most students who shifted towards academic research gave several reasons for doing so, while a small number of students cited individual events, such as the receipt of a large grant or a major publication.

The second trend was that men from well-represented racial/ethnic groups (WR, i.e., White and Asian) appeared to be more likely than any other group to intend to pursue academic research from the beginning to graduation. For the increasers, the proportion of WR women and UR men was similar. The substantial population of increasers, made up of women from various racial/ethnic groups, differs from what has been reported in previous research.

For other careers types, some emergent patterns resembled those in the academic research category, while others differed. Table 1 shows about half of those intending to pursue teaching-intensive academic careers at graduation were consistent from start to finish, with fewer students fluctuating or shifting towards this career. For industry research, only 2 of the 22 who graduated with this intention were consistent from start to finish, with more students fluctuating or increasing closer to graduation. One of the more dramatic changes over time was the increase in the number of students intending to remain in science careers with non-research roles, such as science writing or policy. By graduation, 25 students had selected into this option though none had expressed that intention consistently from the beginning.

Those who decided to pursue careers in non-research science often utilized career development resources at their institutions or pursued networking opportunities at a late stage in their graduate careers. For example, Megan (pseudonym), a white woman who accepted a science writing position during her graduation year, reported that she learned about science and medical writing through a campus career development program. She had ruled out academic research after her sixth year in the PhD, stating,

“You have to give so much of yourself and so much time…The likelihood that you are going to get that job is pretty low and there’s so much fight. I have no fight left in me at the end of my PhD, let alone for another 30 years.”

Unsure of her path after ruling out an academic career, she realized in the last year of her PhD that she enjoyed and excelled at writing, even though she had not considered that career up until that point. She commented,

“I really wasn’t thinking science writing or medical writing, because writing intimidated me…I was really worried about writing my dissertation. I felt like writing was always going to be like college when you had to write a term paper and you put it off and finally you had to pull an all-nighter and write a crappy paper… But after writing my dissertation, I realized that I didn’t hate it. I liked it, I liked thinking through the ideas. I liked messing around with my sentences. I liked coming up with clear ways to discuss things…I realized maybe I am a good writer, [and] my boss has said I’ve done well.”

Several experiences led Megan to differentiate into science writing as a career. She experienced science writing as distinct from other writing; learned she could apply scientific thinking to a non-research career; and received external validation and support from her advisor for this choice.

Finally, the number of students without a single primary career intention fluctuated during training, but by graduation this number was similar as at the start. UR women were the most likely to want to pursue non-research science or to be non-differentiated and keep their options open at graduation, although the group included students from all genders and racial and ethnic backgrounds. Among the non-differentiated group, academic research remained a potential career for 11 of the 35.

Those considering more than one career at graduation were not so much indecisive as they were open to more than one option and exploring multiple opportunities. For example, David (pseudonym), a white man, was unsure about an academic research career but maintained an interest in bench research. At the end of the PhD, he accepted a postdoc in industry, but was not yet committed to a research career in industry. His long-term options included the management track in industry or a research career in academic or another sector. He reasoned,

“I was looking primarily at non-academic positions, because I have experience with academic positions, and I couldn’t really decide what I wanted to do for a career until I had experience in other areas. I was looking primarily at industry because I’ve always been slightly interested in the industry style of research, mostly because I wouldn’t have to write grants or figure out what to study. It would just be like, ‘here, study this.’ I was like, ‘alright, cool, I can do that.’”

These results caution against overstating the significance of group differences at the expense of acknowledging how individuals from most groups are pursuing various career types.

### Timing of career differentiation

**Fig 2:**
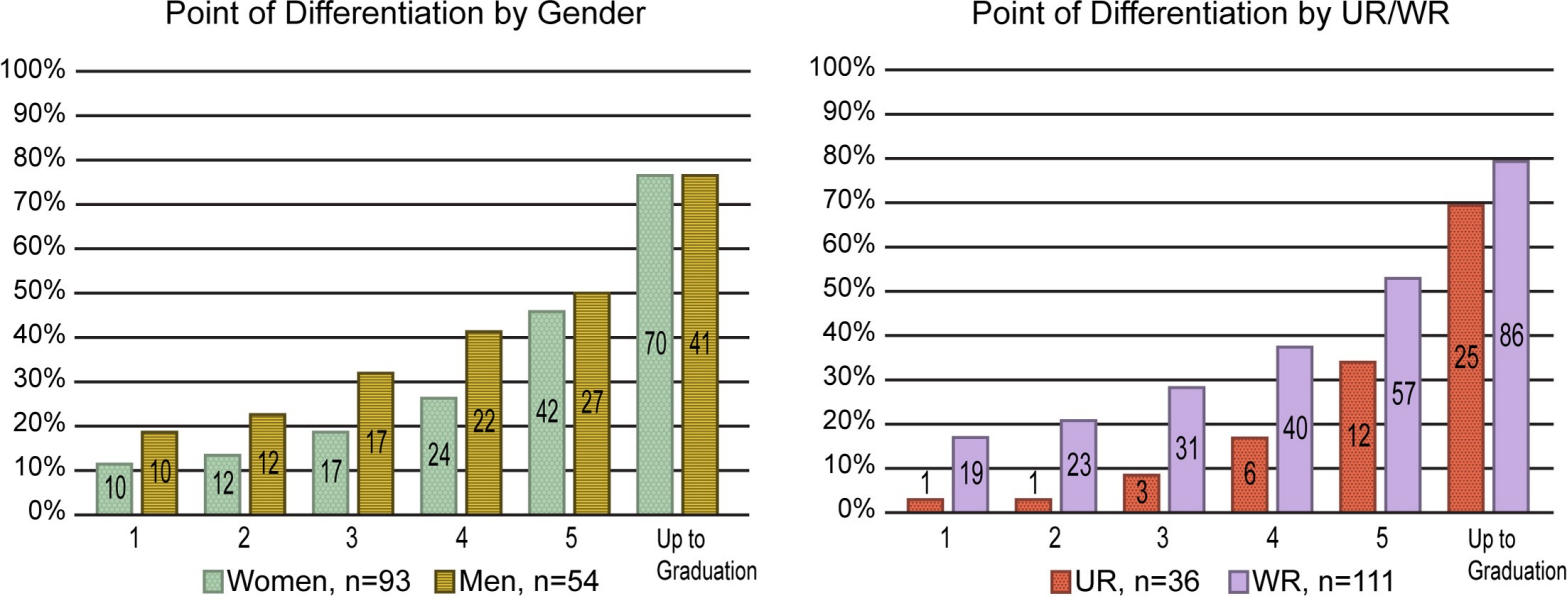
Point of career differentiation during the PhD. The data in Fig 2 represent the “point of differentiation” into a primary career among participants separated by gender and UR/WR status. The point of differentiation is the PhD year at which a participant decided on a career choice, after which they did not waver. The data in the bars are cumulative and display the total number and percentage of participants in each population who differentiated at or before a specified time point. The start of the fifth year of the PhD was the first year in which anyone in the study graduated and is the last real time point reflected. The denotation “up to graduation” captures any differentiation that occurred between the end of the fifth year and graduation. While there were no significant differences (Pearson’s chi-squared test, p < .05) between men and women, a significantly smaller proportion of UR trainees differentiated into a career during the first three years when compared to WR participants.

Fig 2 illustrates the cumulative processes of differentiating into primary career intentions during graduate school. It displays the point of differentiation by gender and UR/WR status. For most groups, fewer than half the participants differentiated before the 5th year, and 20%-30% had not decided on a primary career intention at graduation. Remaining undifferentiated at graduation was quite common across the sample, though there was some variation across groups. For example, although not statistically significant, men appeared to be more likely than women to differentiate early in the PhD, but by graduation, an equivalent proportion (~75%) of men and women had differentiated. There were more apparent differences between WR and UR students. A higher proportion of WR than UR participants differentiated at each interval up to the 5th year of the PhD. For each of the first 3 years, these differences were statistically significant (Pearson’s chi-squared test, p < .05). By graduation, the apparent difference had diminished somewhat and was no longer statistically significant. Despite this, a lower proportion of UR students overall had differentiated before graduation.

When comparing differentiation across all types of careers and demographic groups, the data show only modest apparent differences, none of which rose to statistical significance. As mentioned, WR men appeared to be most likely to choose academic research careers. No UR women expressed an intention to pursue industry research at graduation, and they were most likely to pursue non-research science or to be non-differentiated at graduation. Across career categories, with the exception of academic teaching, participants were more likely to shift in their intentions than to remain unchanged from the beginning. These results caution against overstating the significance of broad group differences at the expense of examining how individuals from most groups are spread across most career types.

### Ruling-out academic research

**Fig 3:**
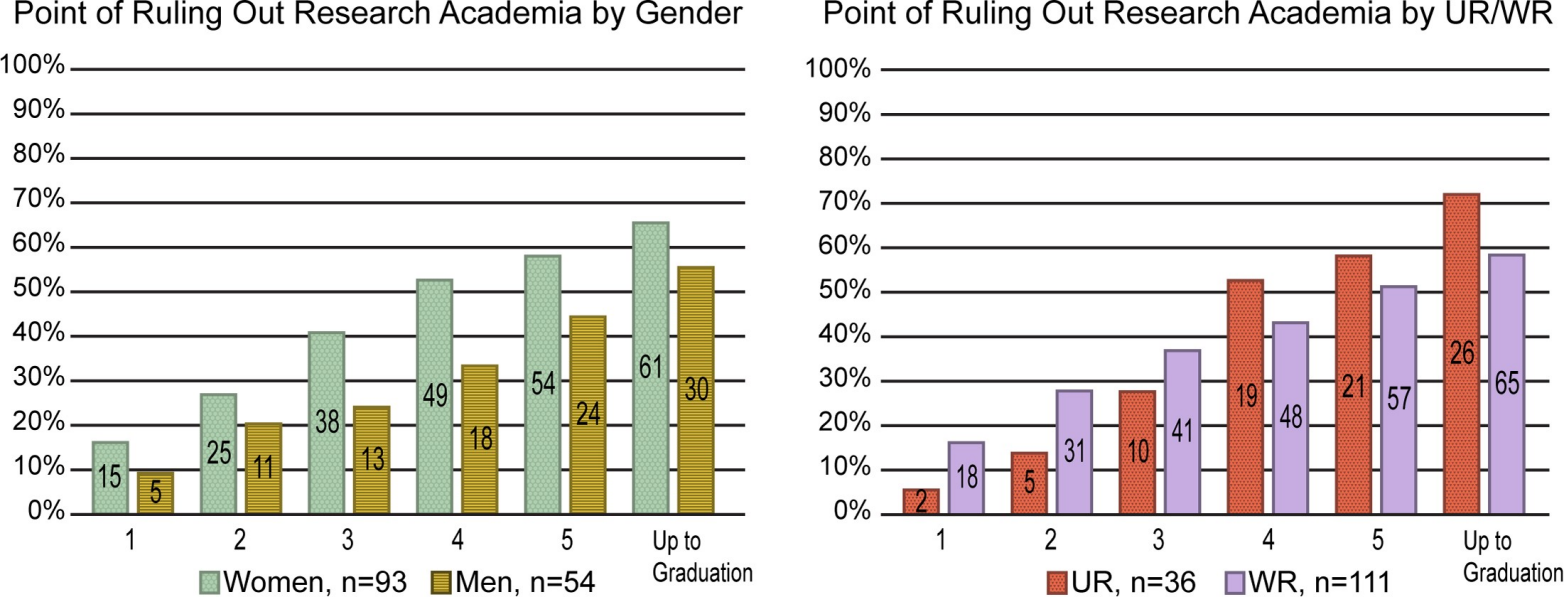
Point of ruling-out an academic research career. Fig 3 shows data for the 91 individuals who ruled out an academic research career during their PhD training by gender and UR/WR status. The charts illustrate the year in which a participant ruled out this career option. The data reflected in the bars are cumulative and display the total number and percentage of participants from each population who ruled out an academic research career at or before the time point indicated. The start of the fifth year of the PhD was the first year in which anyone in the study graduated and is the last real time point reflected. The denotation “up to graduation” refers to any decision-making that occurred between the end of the fifth year and graduation. Note, this figure reflects subjects who definitively ruled out academic research and does not include anyone who had an academic research career as a mixed top choice at graduation. There are no significant differences (Pearson’s chi-squared tests, p < .05) between men and women nor between UR and WR participants as to when an academic research career was ruled out.

At the point of graduation, 39 participants intended to pursue an academic research career. Eleven who were non-differentiated were still considering it as one of their options. The data in Fig 3 reveal the time points at which the remaining 91 students ruled out an academic research career. These 91 include those who started the PhD with no interest in an academic research career and those who ruled it out at some point during their training. Within this sample, decline in intention towards academic research careers occurred gradually; overall, shifts away from academic research were not more or less common at any particular time point. Proportionally, UR students and women were somewhat more likely than WR students and men to rule out academic research careers. The decline in intention for both groups occurred over time and not at discrete points early in PhD programs.

## Discussion

These results reveal the highly dynamic and fluid processes for biomedical PhD students as they differentiate into careers from the beginning of the PhD through graduation. Upon arriving at an intended career, most students will have gone through an extensive process of change or fluctuation in their career intentions before differentiating. While some students held on to the same career intention from start to finish, they were the exception. Taken together, this suggests that graduate school is more likely to be a time of discovering a preferred career than following one career goal from start to finish.

Previous studies have reported an overall decline in interest in academic research careers during the PhD. However, a net decrease in interest was not visible when we compared the number of students intending to pursue academia in their first year and at graduation. The number of students differentiating into academic careers by graduation was similar to the number intending to pursue academic careers at the beginning, although many of the individuals were different. This important new observation could only have been found by longitudinally following the career intentions of each person. Additionally, our study did not observe the general decline in interest in academic research careers early in graduate training reported in previous research. Instead, our data suggest a gradual decline over the course of PhD training. Thus, national-level trends do not tell a complete story about fluctuations in career intentions. By observing each student over time, it becomes even more evident how critical it is to understand the various factors that may contribute to shifts towards academic research or any other careers. These shifts toward careers are as important as other patterns, including decline away from careers and persistence in intentions. Based on our results, we caution against relying on surveys revealing broad group differences without examining how individuals from most gender and racial/ethnic groups are represented across all career patterns. Our findings show that graduate school is more likely to be a time of discovering a preferred career than of following one career goal from start to finish.

We observed some variation across gender and racial/ethnic groups in their career intention patterns, though these trends were modest and nuanced. For example, WR men appeared to be most likely to be consistent in their intentions to pursue academic research careers, which aligns with previous research. Nevertheless, a full 2/3 of those who graduated with an academic career intention had shifted towards it or fluctuated only to return to it at graduation. Critically, the population of those shifting toward academic careers comprised women from diverse backgrounds, a trend which contrasts with expectations set forth by previous research. Though the current numbers are small, the 5 UR women who differentiated into academic research arrived at their intention gradually. These 5 women comprise 20% of the population of UR women in the sample. Certainly, as the data show, some students ruled out academic careers, but the population of “increasers” overall suggests more potential than expected for promoting and improving diversity in the academic research workforce. Students from all demographic groups intended to pursue academic research careers at graduation.

Some observations, including the higher proportion of UR women pursuing non-research careers and the tendency for men to differentiate earlier in the PhD, are consistent with previous studies. Our statistically significant finding that UR students differentiated later in their graduate careers than WR students is an area for future investigation. There are many potential explanation for such an observation, such as fewer role models in any field from which to gauge potential futures, or more challenges finding mentors as has been documented previously. However, at present, there are no existing data on how the timing of career decisions during graduate school impacts career success. Thus, no inferences should be drawn as to the relative benefits of early vs. late differentiation.

The current results show that the choices of individuals from across demographic groups do not always align with generalized trends. Curricular or programmatic changes around career mentoring and development based only on broad national trends will miss differences among individuals. These must be tailored toward and support students’ individual experiences and the likelihood of frequent changes during training. To move forward with diversity and career development efforts, it is essential to recognize that students from similar gender and racial/ethnic backgrounds are widely varied in their scientific identities and decision-making. At the individual level, effective advising will require that mentors are not only supportive and knowledgeable of a wide variety of career options, but also sensitized to the potential for trainees to shift in their intentions before arriving at their best-fit careers. Trainees will benefit from mentors who take on a sponsoring role by encouraging their mentees to explore various careers and by facilitating broad networking connections.

## Conclusions and directions for future research

Our work raises a central question for future research. A critical next step in this research is continuously tracking the post-PhD career outcomes of students, and assessing whether their plans continue to align with their intentions at graduation. This study also sets up future analyses of the factors influencing career choice, change, and persistence. How does identification with a career or the roles expected in a career contribute to differentiation? For instance, if most students shift towards an intended career during graduate school, then some process of identification is taking place during this time. Others have pointed to identification with the values and roles of a profession as the main factor leading to career decisions [19]. Yet, we still know very little about the mechanisms, or the processes, of identification and differentiation as they occur over time. This remains an important area for investigation, especially with qualitative data. Arriving at a career intention may be explained by a number of factors, including learning and adapting to the roles related to a career, the perception that a career is achievable, effective mentoring, or even adverse experiences leading students to reconsider their career intentions. The fluidity of career intentions supports the importance of providing effective tools and guidance as students navigate their choices, with the likely implication that effective use of resources will improve the speed and accuracy of decision-making and lead to less attrition from the academic research workforce.

Another question is whether the timing of career differentiation is associated with success in that career. WR men are more likely to be persistent from an early stage in their intentions toward academic careers, but the importance of early identification and persistence in predicting success in academic research or any other career is unknown. Ongoing interviews and analyses will enable person-by-person identification of the factors promoting and impeding progression of young biomedical scientists into an array of careers.

The analysis presented here represents the “what” in the “what, why, and how?” of career decision-making in biomedicine. This report sets the stage for continuing analyses of rich interview data gathered at the individual level during the PhD, and ongoing data gathered after degree completion. Forthcoming analyses will delve more deeply into the “why” and “how” of career outcomes, illuminating the mechanisms explaining both decision-making and outcomes. Questions that will be addressed in the future include:

- *Why do students change their career intentions*? *Is it due to awareness of new options or pivotal events*, *which may be idiosyncratic for each student*, *and/or reflective of the culture of a career*? *Do the factors influencing change vary by gender and/or race/ethnicity*?
- *What role do mentors and/or other faculty play in shaping career intentions*?
- *What happens after the PhD with career intentions*? *Do changes reflect evolution in career alignment and identification or responses to pivotal events*?
- *How malleable are career decisions*? *When designing interventions to enable success in a particular career*, *how should these be positioned within the career decisions timeline*, *and what skills*, *competencies*, *and resources should be emphasized*?
- *Among early career academic faculty*, *do factors contributing to success vary by gender and/or race/ethnicity*?

## Supporting information

S1 Dataset (XLSX)

S1 Fig (TIF)
