# Supplementary figures and images for: The National Longitudinal Study of Young Life Scientists: Career differentiation among a diverse group of biomedical PhD students
Source: PLoS One. 2020 Jun 9;15(6):e0234259. doi: 10.1371/journal.pone.0234259 (PMC7282657; doi:10.1371/journal.pone.0234259)

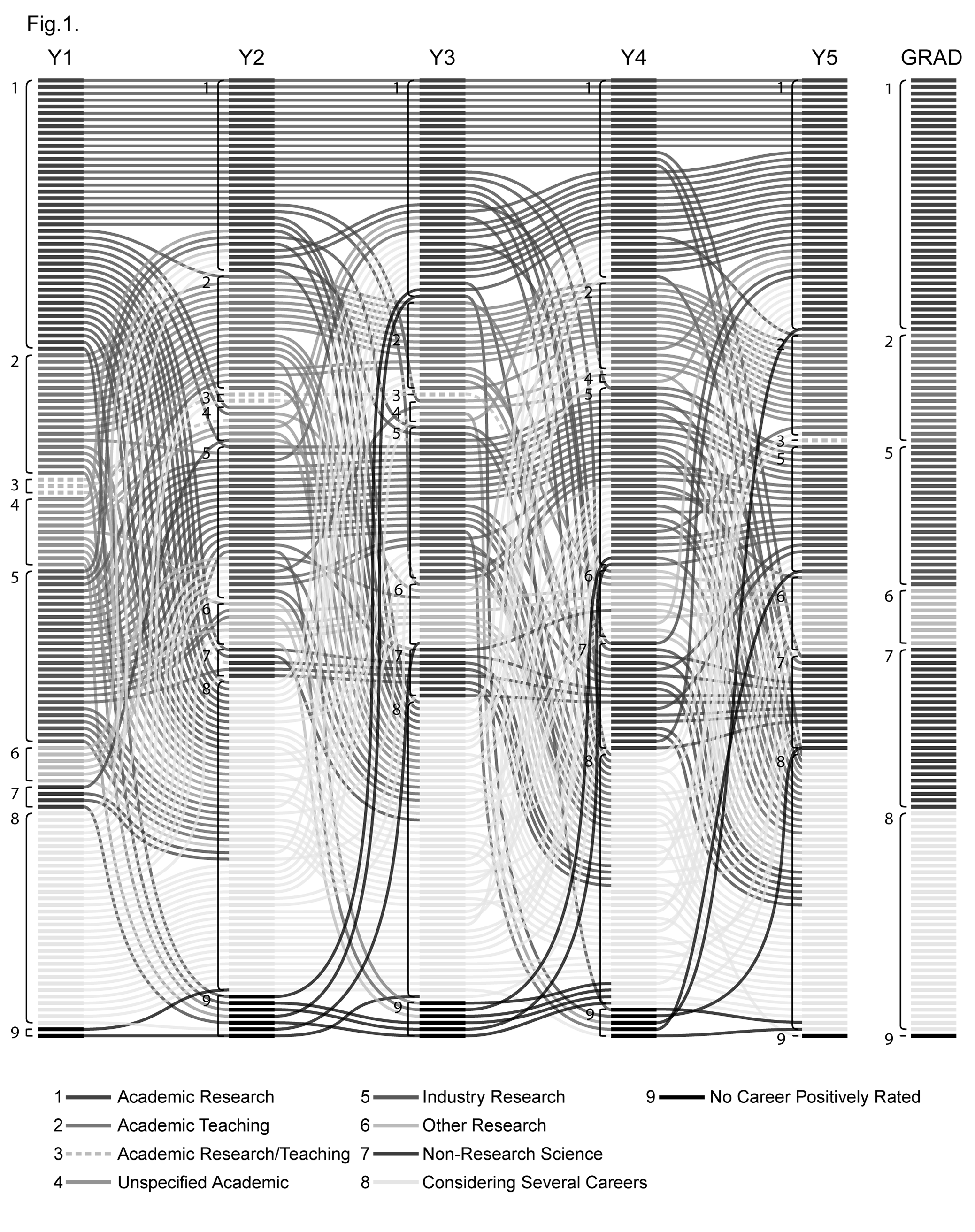

Supplement: S1 Fig — (TIF) [file pone.0234259.s002.tif]
